# Formation of High-Order Oligomers by a Hyperthemostable Fe-Superoxide Dismutase (tcSOD)

**DOI:** 10.1371/journal.pone.0109657

**Published:** 2014-10-14

**Authors:** Sha Wang, Zhi-Yang Dong, Yong-Bin Yan

**Affiliations:** 1 State Key Laboratory of Microbial Resources, Institute of Microbiology, Chinese Academy of Sciences, Beijing, China; 2 State Key Laboratory of Biomembrane and Membrane Biotechnology, School of Life Sciences, Tsinghua University, Beijing, China; Aligarh Muslim University, India

## Abstract

Hyperthermostable proteins are highly resistant to various extreme conditions. Many factors have been proposed to contribute to their ultrahigh structural stability. Some thermostable proteins have larger oligomeric size when compared to their mesophilic homologues. The formation of compact oligomers can minimize the solvent accessible surface area and increase the changes of Gibbs free energy for unfolding. Similar to mesophilic proteins, hyperthermostable proteins also face the problem of unproductive aggregation. In this research, we investigated the role of high-order oligomerization in the fight against aggregation by a hyperthermostable superoxide dismutase identified from Tengchong, China (tcSOD). Besides the predominant tetramers, tcSOD could also form active high-order oligomers containing at least eight subunits. The dynamic equilibrium between tetramers and high-order oligomers was not significantly affected by pH, salt concentration or moderate temperature. The secondary and tertiary structures of tcSOD remained unchanged during heating, while cross-linking experiments showed that there were conformational changes or structural fluctuations at high temperatures. Mutational analysis indicated that the last helix at the C-terminus was involved in the formation of high-order oligomers, probably via domain swapping. Based on these results, we proposed that the reversible conversion between the active tetramers and high-order oligomers might provide a buffering system for tcSOD to fight against the irreversible protein aggregation pathway. The formation of active high-order oligomers not only increases the energy barrier between the native state and unfolded/aggregated state, but also provides the enzyme the ability to reproduce the predominant oligomers from the active high-order oligomers.

## Introduction

Hyperthermostable enzymes are usually isolated from hyperthermophiles grown at extremely high temperatures [1]. Although hyperthermostable enzymes have very similar primary sequences and tertiary structures to their mesophilic homologues, they show remarkable stability against denaturation induced by heat or chemical denaturants [2]. Many structural stabilizing factors have been proposed to contribute to the extreme thermostability of soluble hyperthermostable proteins, such as stronger ion-pairing network, additional disulfide bridges, higher order oligomerization, conformational rigidity, fewer cavities and more compact structures when compared to the mesophilic proteins [1]–[7]. In most cases, hyperthermostable proteins use most of these structural stabilizing factors to fight against the extreme conditions, which makes it difficult to provide a unified explanation of protein hyperthermostability [2]. The case is much more complex in multimeric proteins than in monomeric proteins due to additional subunit interactions and spatial organization in their quaternary structures. The characterization of the molecular origin of protein hyperthermostability can not only facilitate the applications of important industrial enzymes, but also help us to understand the key factors in enzyme evolution and protein stability.

Compared to their mesophilic homologues, some hyperthermostable proteins have larger oligomeric sizes [1]. The formation of proper oligomeric structures is crucial not only to the catalysis of the hyperthermostable enzymes at extreme temperatures, but also to their hyperthermostability [1], [8]–[14]. The selection of oligomeric proteins by hyperthermophiles may be caused by the extra stabilizing factors provided by subunit interactions. Consequently, destroying the oligomeric structures by truncations or point mutations significantly decreased the stability of hyperthermostable enzymes against heat or chemical denaturants [10], [11], [13], while destabilizing the monomers seems to have a minor effect on the stability of oligomeric hyperthermostable enzymes [15], [16]. The importance of oligomerization has been observed not only in proteins from hyperthermophiles, but also in some long-lived ultrastable proteins and molecular chaperones in vertebrates such as crystallins [17]–[20]. Some molecular chaperones have been characterized to be able to change their oligomeric states in response to stresses, and the conversion among different oligomeric states is proposed to be closely related to their physiological functions under various conditions [21]–[27]. However, the studies of alternations in the oligomeric states and their implications in structural stability are rather limited for hyperthermostable proteins.

Superoxide dismutases (SODs) are evolutionally highly conserved enzymes that catalyze the disproportionation of superoxide radicals [28]. By modulating the superoxide levels, SODs are involved in many physiological and pathological processes [29]–[32] and have important industrial and pharmacological applications [33], [34]. SODs are classified into four types named by their preference of the coordinated metal ions: Cu/Zn-, Mn-, Fe- and Ni-SODs [28], [35]. Fe-SODs are usually characterized from hyperthermophiles living in hotsprings [9], [36]–[38]. TcSOD is a hyperthermostable Fe-SOD cloned from the metagenomic library of hot springs in Tengchong, China [39]. TcSOD shares a high homology to Fe-SOD from *A. Pyrophilus* (ApSOD) [37], [39], [40]. Similar to ApSOD, tcSOD mainly exists as a compact tetramer in diluted solutions [12], [39]. In this research, we found that tcSOD can associate into active higher-order oligomers larger than tetramer. A mutational screening indicated that the removal of the last 10 residues (M202) at the C-terminus greatly decreased the ability of tcSOD to form high-order oligomeric structures. The mutant M202 also possessed a lower thermostability and aggregation-prone property, implying that the formation of high-order oligomers might be important to tcSOD hyperthermostability.

## Materials and Methods

### Materials

Tris, dithiothreitol (DTT), dithiobis(succinimidylpropionate) (DSP), isopropyl-1-thio-β-d-galactopyranoside (IPTG), glutaraldehyde and sodium dodecyl sulfate (SDS) were purchased from Sigma Chemical Corporation. The vector pET28a was purchased from Novagen. All other chemicals were local products of analytical grade.

### Protein expression and purification

The cloning of the genes of wild type (WT) tcSOD and two truncation mutants, M202 (residues 1–201) and M211 (residues 1–210) has been described elsewhere [16], [39]. The point mutation mutants were obtained using standard site-directed mutagenesis method. The mutagenic primers were: H4D-forward, 5′- 5′-CGGGATCCATGCCAGTGGATAAGTTA-3′; K12A-forward, 5′-CGGGATCCATGCCAGTGCATAAGTTAGAGCCAAAGAACCATCTTGCGCCTTC-3′; K12A-reverse, 5′-CCAAGCTTTTATTTCACAAAATCCTTGAGGGTTTCATAAGC-3′; E23A-forward, 5′-CATTTCCAACGCGCAGATTGAG-3′; E23A-reverse, 5′-GGCTCAATCTGCGCGTTGGAAAT-3′; E165A-forward, 5′-ACTTACGCACACGCCTAC-3′; E165A-reverse, 5′-AGTAGGCGTGTGTGCGTAAGTGT -3′; E165 V-forward, 5′-ACTTACGTACACGCCTAC-3′ and E165 V-reverse, 5′-AGTAGGCGTG TACGTAAGTGT-3′. The WT and mutated proteins were overexpressed in *E. coli* BL21 induced by IPTG and purified by Ni-NTA affinity chromatography as described previously [16], [39]. The final products were dissolved in 20 mM Tris-HCl buffer or 20 mM phosphate buffered saline (PBS), pH 7.4, with the addition of 0.05 mM DTT. The protein concentration was determined by the Bradford method [41].

### Electrophoresis and activity assay

The 12.5% SDS-PAGE and 10% native-PAGE of the proteins were conducted according to the standard methods. The 10% native-PAGE was run using the protocol similar to that of SDS-PAGE except for the absence of SDS in the loading buffer and the gel. SOD activity was assayed according to the standard pyrogallol autoxidation methods [42]. The SOD activity was defined as the amount of enzyme that inhibits the autoxidation of pyrogallol by 50% [42]. SOD activity was also assayed by staining the 10% native-PAGE gel using the nitro blue tetrazolium reaction as described previously [43].

### Spectroscopic experiments

Spectroscopic measurements were performed at 25°C using a protein concentration of 0.27 mg/ml in 20 mM Tris-HCl buffer, pH 7.4 as those described elsewhere [12]. In brief, the far-UV circular dichroism (CD) spectra were measured on a Jasco 715 spectrophotometer and the fluorescence spectra were measured on an F-2500 spectrophotometer with an excitation wavelength of 295 nm.

### Chemical cross-linking

Cross-linking by glutaraldehyde was performed according to the method described previously with some modifications [12]. In brief, 100 µl protein solutions with a protein concentration of 0.27 or 1 mg/ml were heated at a given temperature (from 25°C to 80°C) for 5 min or 50 min, followed by mixing with 15 µl freshly prepared glutaraldehyde solutions (2.5%) preheated at the same temperature. The reaction was performed in 20 mM sodium phosphate buffer, pH 7.4. After cross-linking, the reaction was terminated by the addition of 15 µl preheated Tris-HCl stock solutions (1 M, pH 8.0). Cross-linking by DSP was carried out by equilibrated 50 µl protein solutions in the absence of DTT at a given temperature for 50 min, and then 3 µl DSP stock solutions was added to reach a final DSP concentration of 0.5 mM. After cross-linked by DSP for 10 min, 3 µl Tris-HCl stock solutions (1 M, pH 8.0) were added to terminate the reaction. Bovine serum albumin (BSA) was used as a control for the cross-linking experiments. The cross-linked proteins were analyzed by 10% native-PAGE and 12.5% SDS-PAGE.

### Separation of high-order oligomers

The separation of the tetramers and high-order oligomers was performed by ion exchange using the 1 ml Resource Q column equipped on an ÄKTA FPLC (Amersham Phamacia Biotech, Sweden). The column was equilibrated with 20 mM PBS. About 2 ml protein solutions with a concentration of 1 mg/ml was injected in the column and eluted with a linear gradient of 0–500 mM NaCl in 20 mM PBS.

### Size-exclusion chromatography

The size-exclusion chromatography (SEC) experiments were carried out on a Superdex 200HR 10/30 column on an ÄKTA FPLC. The column was pre-equilibrated with 20 mM sodium phosphate buffer (pH 7.4), and then 100 µl protein solutions were injected into the column. All samples were run at a flow rate of 0.5 ml/min at 25°C.

## Results

### Oligomerization of tcSOD

**Figure 1:**
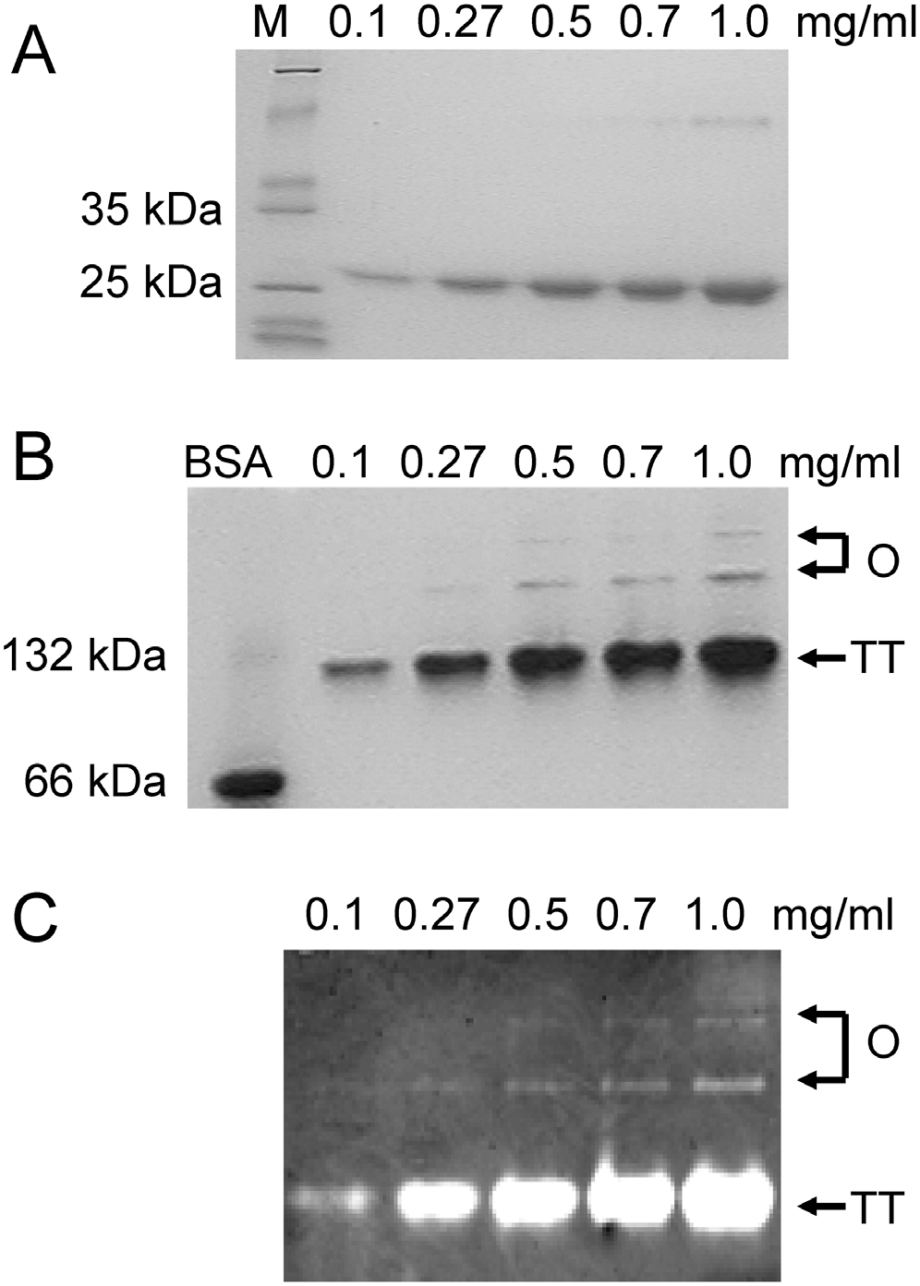
Protein-concentration dependence of high-order oligomerization. (A) 12.5% SDS-PAGE. (B) 10% native-PAGE. (C) Activity staining of the bands in native-PAGE. M is the protein marker, TT is tetramer and O is high-order oligomers. The proteins were dissolved in 20 mM PBS buffer, pH 7.4.

We evaluated the oligomeric equilibrium of tcSOD by SEC and spectroscopic analysis by varying protein concentrations. The elution volume and spectroscopic characteristics remained unchanged for protein concentration ranging from 0.1 mg/ml to 1.0 mg/ml (Figure S1 in File S1), consistent with the previous finding that tcSOD mainly exists as a compact tetramer [12], [39]. The oligomeric states of tcSOD were further investigated by 10% native-PAGE gel and 5–15% gradient native-PAGE, which can sensitively probe the minor fractions. Both gels provided similar results, and the gel of 10% native-PAGE is presented in Figure 1. BSA, which mainly exist as a monomer and contains a small portion of oligomers, was used as the control and molecular weight reference [44]. Besides the predominant tetramer band, a minor amount of high-order oligomers, which were octamer (dimer of tetramer) and above, could be identified at higher protein concentrations. Quantitative analysis by the by GIS software (Tanan Company, China) indicated that the fraction of the high-order oligomers increased from 1.5% to ∼5% when the protein concentration was increased from 0.1 mg/ml to 1.0 mg/ml. These large oligomers were fully active as reflected by the activity staining using the nitro blue tetrazolium method [43].

**Figure 2:**
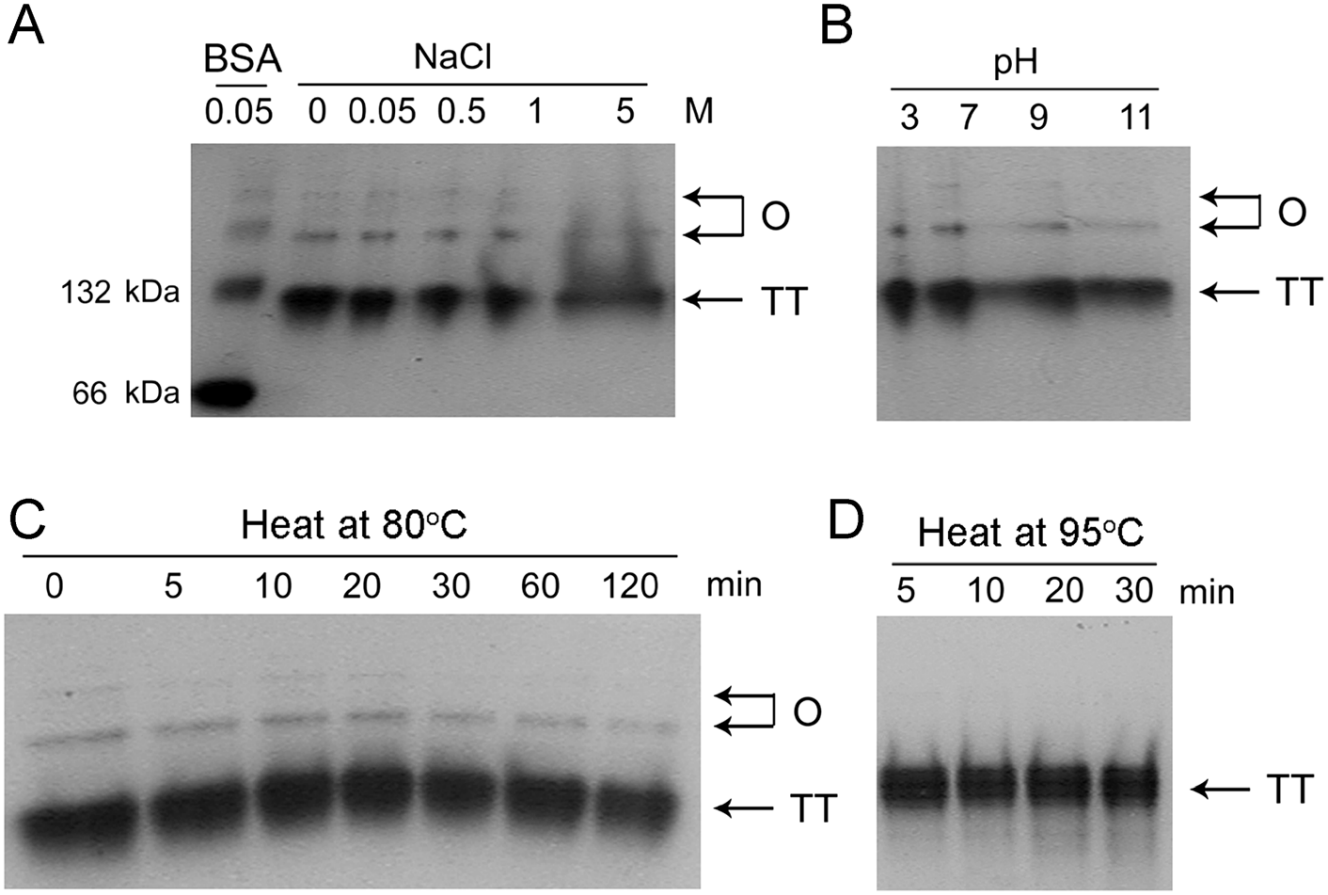
Effects of solution conditions on the dynamic equilibrium between tetramer and high-order oligomers characterized by 10% native-PAGE. (A) Salt-dependence. (B) pH-dependence. (C) Heat treatment at 80°C. (D) Heat treatment at 95°C. The proteins were dissolved in 20 mM PBS buffer with a protein concentration of 1 mg/ml. TT is tetramer and O is high-order oligomers. BSA was used as a molecular weight reference and a loading control of the native-PAGE. For clarity, the BSA band was not shown in panels B, C and D.

The effect of environmental factors on the conversion among various oligomeric states was investigated by incubating the proteins in various solutions and analyzed by native-PAGE. The results in Figure 2 indicated that the fractions of the high-order oligomers remained almost unchanged when altering the ion strength by adding 0 M to 5 M NaCl, varying pH from 3 to 11 or heating at 80°C for 60 min. The high-order oligomers were disappeared after 5 min treatment at 95°C, a temperature at which the enzyme began to be inactivated by heat treatment [16], [39]. These observations implied that the formation of high-order oligomers was more likely to be driven by hydrophobic but not electrostatic interactions.

**Figure 3:**
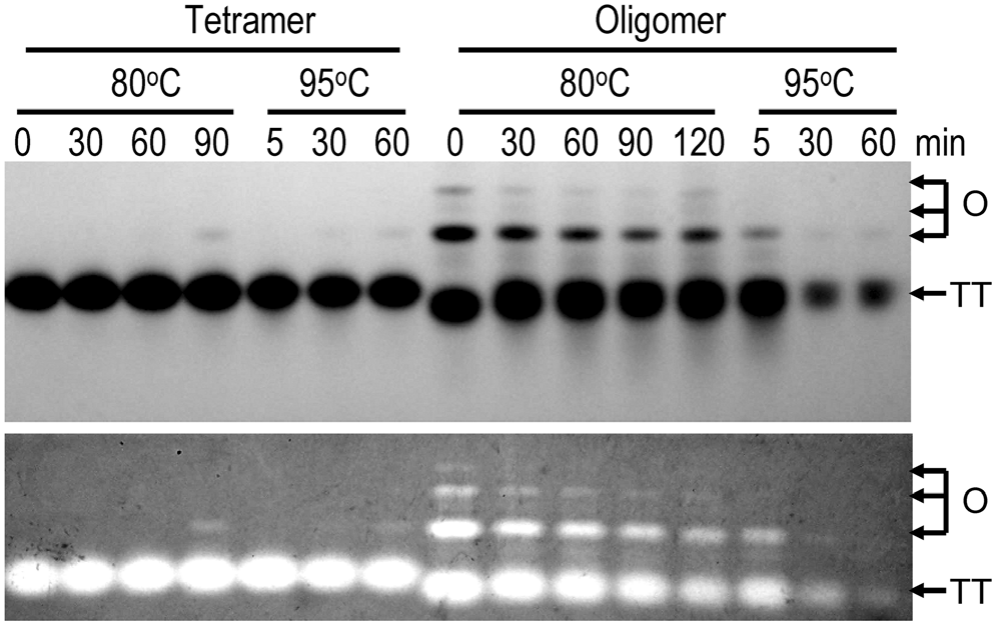
Stability of purified tetramers and high-order oligomers separated by ion-exchanging. Most of the high-order oligomers were quickly dissociated into tetramers after collection. The purified fractions were concentrated to a protein concentration of 0.3 mg/ml and heated at 80°C or 95°C for a given time. After heat treatment, the samples were analyzed by 10% native-PAGE (upper panel) followed by activity staining (lower panel). A weak band from octomer can be observed when the tetramers were heated at 80°C for 90 min or 95°C for 60 min. TT is tetramer and O is high-order oligomers. The oligomeric size was characterized by the position of the native tetramer as well as the BSA control (data not shown).

To further characterize the roles of high-order oligomers in tcSOD hyperthermostability, the tetramers and high-order oligomers were separated by ion exchange using the Resource Q column. Most of the separated high-order oligomers quickly dissociated into tetramers (Figure 3), reflecting that there was a dynamic oligomeric equilibrium between tetramers and large oligomers. Consequently, a small fraction of octamers could be induced when the tetrameric proteins were heated at 80°C or 95°C, and higher temperature could accelerate the conversion process. The high-order oligomers were less stable than the pure tetramers or native proteins at high temperatures, and a significant loss of activity could even be observed at 80°C. At 95°C, the amounts of tetramers and large oligomers decreased continuously along with the increase of incubation time due to protein aggregation. Notably, the position of the tetramers dissociated from high-order oligomers was slightly different with the native tetramers, suggesting that a conformational change was necessary for the conversion between tetramers and large oligomers.

### The C-terminus of tcSOD contributes to the formation of high-order oligomers

**Figure 4:**
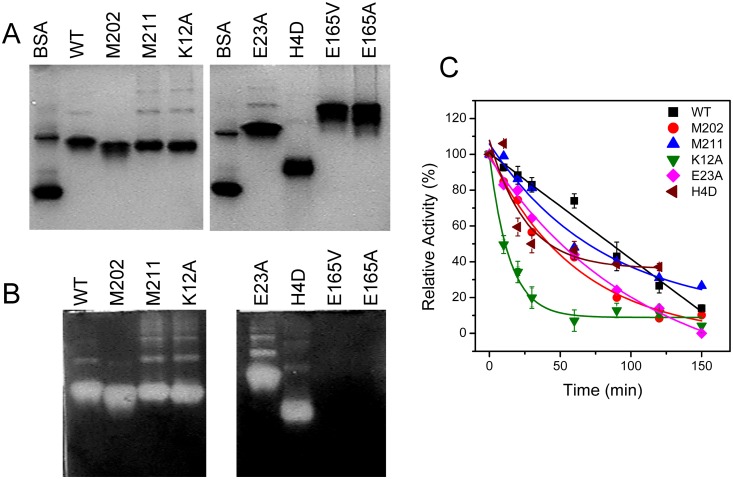
Effect of mutations on the high-order oligomerization and stability of tcSOD. (A) Native-PAGE. (B) Activity staining. (C) Stability at 95°C. The protein concentration was 1 mg/ml for the native-PAGE and activity staining analysis and 0.33 mg/ml for the stability experiments. The presented data were calculated from three repetitions.

Mutational analysis was performed to gain insight into the structural basis of tcSOD high-order oligomerization. According to the crystal structure of ApSOD [40] and the modeled structure of tcSOD [39], several key residues involved in the intra- or inter-subunit ion-pairing network were selected and the recombinant mutated proteins were produced by the *E. coli* overexpression system. Among the mutations, H4D, E23A, E165A and E165 V were expected to destroy the ion-paring network at the subunit interfaces, K12A was designed to destroy intra-subunit ion-pairing network, while M202 and M211 were two mutants with the removal of 10 residues or 1 residue off the C-terminus, respectively. The purified E165A and E165 V proteins were inactive even though they retained the quaternary structure, while the other mutants had similar activities with the WT enzyme (Figure 4). Thermostability analysis indicated that most mutants could retain more than 90% activity except for K12A exhibiting a significant activity loss when heated at 80°C for 120 min (Figure S2 in File S1). At 95°C, K12A was very unstable and lost 50% activity within 10 min. The other mutants were slightly less stable than the WT enzyme (Figure 4C). The behavior of the K12A mutant in tcSOD was different from the stability-enhancing effect observed in ApSOD [45]. Nonetheless, the comparative native-PAGE and activity staining analysis indicated that among the active mutants, M202 was the only one with the absent of high-order oligomerization. This observation suggested that the helix at the C-terminus might be involved in the formation of large oligomers.

### Conformational change of tcSOD at high temperatures by cross-linking

The above results suggested that there was an energy gap for the conversion from tetramer to high-order oligomers and vice versa, which might be caused by the reorganization of the compact tetrameric molecule to large oligomers. The oligomeric conversion could be accelerated by high temperatures (Figure 3), and thus the conformational changes during heating were investigated by spectroscopic methods and cross-linking experiments. No significant changes were observed in the secondary and tertiary structures when evaluated by far-UV CD, Fourier transform infrared spectroscopy, light scattering and intrinsic Trp fluorescence when the WT protein and M202 were heated from 25°C to 85°C (data not shown). Actually, the structure of tcSOD maintained intact even heated at 85°C or 95°C for up to 200 min (Figures S3 and S4 in File S1). The little alternation in spectroscopic characteristics might be caused by the fast-exchange of various conformational states and the low population of the high-order oligomers.

**Figure 5:**
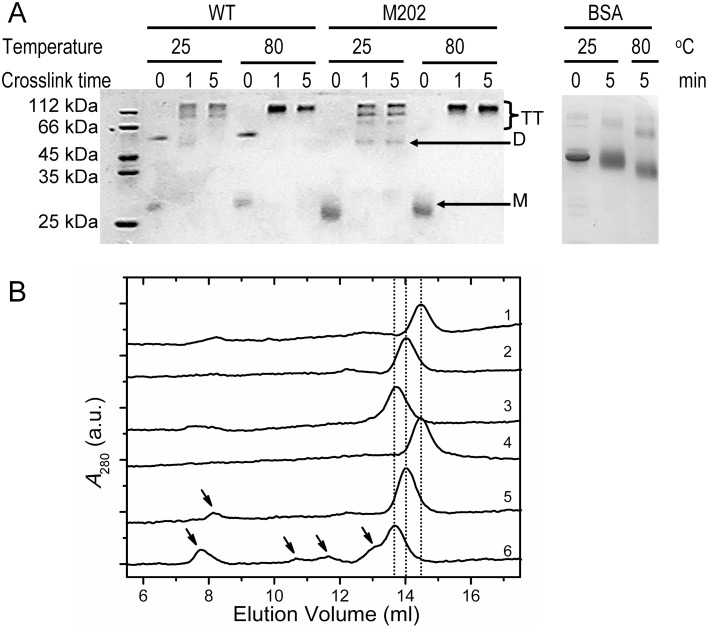
SDS-PAGE and SEC analysis of the proteins cross-linked by glutaraldehyde. (A) SDS-PAGE analysis of the WT and M202 samples with or without cross-linking after heating at 25°C or 80°C for 50 min. The cross-linking was performed at the incubation temperature for 1 min or 5 min. after heat treatment. The right panel shows the SDS-PAGE of BSA treated at the same conditions as a control. M is monomer, D is dimer and TT is tetramer. (B) SEC analysis of the WT TcSOD and M202 cross-linked at 25°C or 80°C. The SEC samples were cross-linked for 5 min after heating at the given temperature for 50 min with a final protein concentration of 0.27 mg/ml. 1–6 represent the WT protein without cross-linking, WT protein cross-linked at 25°C, WT protein cross-linked at 80°C, M202 without cross-linking, M202 in cross-linked at 25°C and M202 cross-linked at 805°C, receptivity. The arrows indicate the peaks from the off-pathway aggregates.

Cross-linking is a sensitive probe to monitor the structural fluctuations and subtle conformational alternations in proteins [46], [47]. Thus cross-linking by glutaraldehyde was performed for proteins heated at temperatures ranging from 25°C to 80°C, and then the cross-linked samples were analyzed by SDS-PAGE, native-PAGE and SEC analysis. SDS-PAGE analysis (Figure 5A) indicated that at 25°C, the bulk of the cross-linked WT tcSOD and M202 molecules were tetramers, and a minor fraction of dimers could also be observed. Two tetrameric bands could be distinguished, indicating that the cross-linked proteins were differ in their molecular shapes due to dissimilar intermolecular cross-linked conformations [48]. When cross-linked at 80°C, only one major tetrameric band could be observed in the SDS-PAGE, which might be caused by the relative higher efficiency of the covalent-bond formation between the protein and glutaraldehyde or conformational changes of tcSOD during heating. When BSA was used as a control, no significant changes in cross-linking efficiency were observed. This suggested that tcSOD might undergo structural change at higher temperatures, though it was undetectable by spectroscopic methods. SEC analysis indicated that cross-linked proteins eluted earlier when compared with the non-treated proteins (Figure 5B). Meanwhile, the proteins cross-linked at 80°C had a relative smaller elution volume than that cross-linked at 25°C. It seems that cross-linking resulted in an apparently larger molecular weight of the protein, which could be caused by the increase of the molecular size since the contribution of the small molecular weight cross-linker could be negligible. Compared with the cross-linked WT protein, there was an additional small peak from aggregates appeared at around the void volume of the column in the SEC profile of M202 when cross-linked at 25°C. The peak from aggregates was much more intense when M202 was cross-linked at 80°C. Besides the peak at around the void volume, several peaks from small aggregates/non-native oligomers could also be distinguished between 10 and 13 ml in the SEC profile of M202 cross-linked at 80°C. Thus it seems that M202 was prone to convert into conformations facilitating protein aggregation.

**Figure 6:**
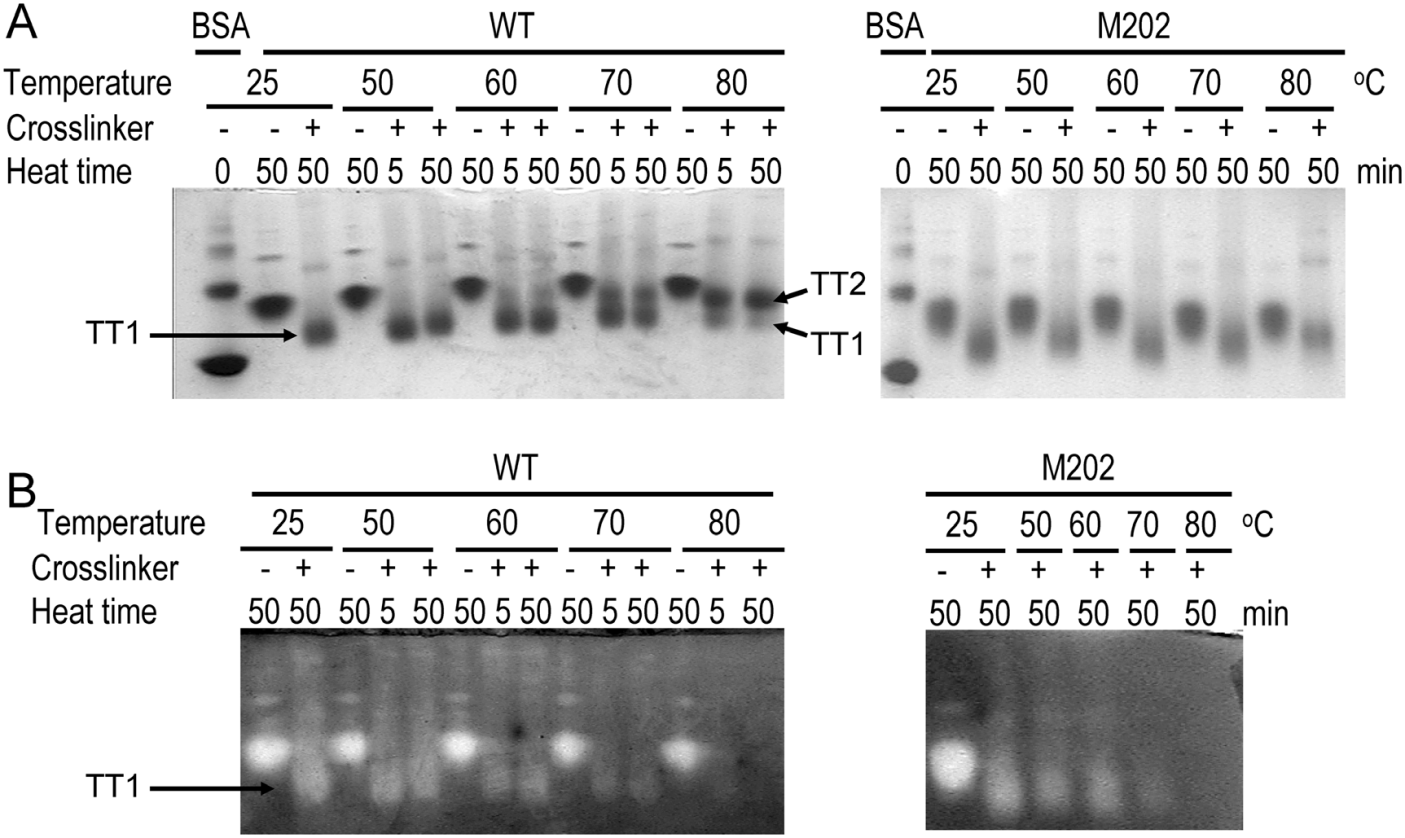
Native-PAGE and activity staining of the proteins cross-linked by glutaraldehyde. (A) 10% Native-PAGE. (B) Activity staining. The proteins were heated for a given time (0, 5 or 50 min) at a given temperature ranging from 25°C to 80°C, and then cross-linked by glutaraldehyde for 10 min at the incubation temperature. TT1 and TT2 indicate two different forms of cross-linked tetramers. TT1 is active and TT2 is inactive. The protein concentration was 1 mg/ml. The sample cross-linked at 80°C contained insoluble aggregates (data not shown).

To further investigate the possible conformational changes and/or oligomeric states alternations at high temperatures, native-PAGE followed by activity staining experiments were performed for samples cross-linked at different temperatures (Figure 6). The bands of the non-crosslinked tcSOD remained unchanged upon heating in the native-PAGE, which coincided with the results shown in Figure 1. At 25°C, the cross-linked tetrameric specie (TT1) had a higher mobility in the native-PAGE, demonstrating that cross-linking led to a change in the solvent-exposed charge, size or molecular shape of TcSOD. In addition to the tetrameric form, parts of the cross-linked proteins existed as large multimers with high dispersion. The amounts of large oligomers seemed to increase when the temperature was above 50°C. However, a quantitative evaluation could not be achieved due to the large dispersion of the large oligomers in the native-PAGE. The cross-linked enzymes were active as indicated by the activity staining of the bands. At temperatures above 60°C, one additional tetrameric band (TT2) appeared with relative smaller mobility in the native-PAGE. Along with the appearance of TT2, the activity of the cross-linked samples decreased and completely lost at 80°C. The inactivation of tcSOD by cross-linking at high temperatures was caused by changes in the glutaraldehyde-accessible sites because the non-crosslinked enzyme was fully active at 80°C. The conversion from active TT1 to inactive TT2 suggested that a conformational change occurred during the heating of TcSOD at high-temperatures. The overall behavior of M202 was similar to the WT protein except for that the TT2 band could not be clearly distinguished and the significant decrease in active high-order oligomers.

### Cross-linked tcSOD dimer is active

**Figure 7:**
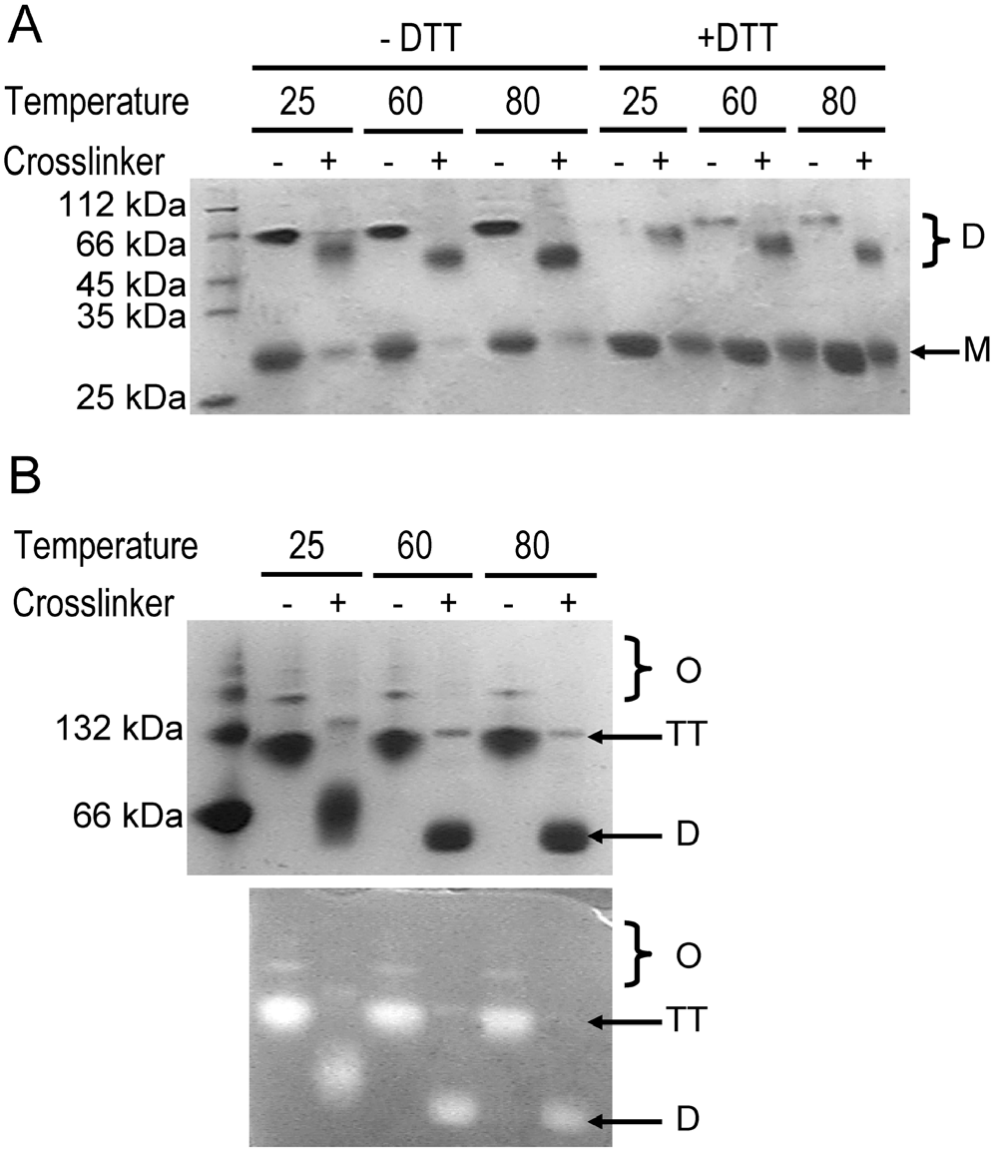
Cross-linking of tcSOD by DSP. (A) SDS-PAGE. (B) Native-PAGE (upper) and activity staining (lower). The samples were incubated at the given temperature for 50 min followed by 10 min cross-linking by DSP. M is monomer, D is dimer, TT is tetramer and O is high-order oligomers.

We also tested another cross-linking reagent DSP (Figure 7). Since DSP contains a disulfide bond within the molecule, the cross-linking experiments were performed in buffer in the absence of reducing reagent. Unlike glutaraldehyde, cross-linking by DSP could only stabilize the dimers. The dissimilarity in the efficiency of the different cross-linkers might be caused by the dissimilar size of the cross-linkers. DSP is much larger than glutaraldehyde, and thus is expected to have less binding sites than glutaraldehyde. Cross-linking of two subunits in a tetramer by DSP could dissociate the tetramer into dimers as revealed by the SDS-PAGE and native-PAGE analysis, suggesting that the cross-linking disrupted the dimer-dimer interface. Interestingly, activity staining indicated that the cross-linked dimers were catalytically active, implying that tetramerization of tcSOD was more likely to be caused by the requirement of hyperthermostability but not catalysis.

## Discussion

Compared with monomeric proteins, oligomeric proteins usually have extra structural stabilizing factors via subunit interactions, which provide not only additional hydrophobic and electrostatic interactions at the subunit interfaces but also conformational constraints of the motions of the subunits. Consequently, some hyperthermophilic proteins have evolved to adopt oligomeric forms rather than monomeric form [1]. However, oligomeric proteins are still facing the problem of misfolding and aggregation under extreme conditions. Notably, aggregation is an irreversible process that usually leads to loss-of-function of proteins and may be toxic to the cells [49]. It is of great interest to investigate the molecular mechanism for hyperthermostable proteins to fight with the off-pathway processes under the extreme high temperature.

The importance of the dynamic oligomeric equilibrium has been characterized in many mesophilic proteins including the ultrastable molecular chaperones. The conversion among oligomeric states of these proteins is usually associated with functional switch. One example of stability-linked oligomerization is β-crystallins, which are long-lived stable homomers and heteromers with 2–8 subunits depending on protein concentrations and environmental conditions [19]. Disrupting the dynamic oligomeric equilibrium of β-crystallins by inherited mutations has been proposed to contribute to the onset of congenital cataract [18], [20]. Another example is the ultrastable monomeric enzyme ribonuclease A, which can form active domain-swapped oligomers under extreme conditions such as high temperatures and lyophilization [22]. Thus it seems that the formation of active high-order oligomers may be a general mechanism for proteins to achieve ultrastability.

**Figure 8:**
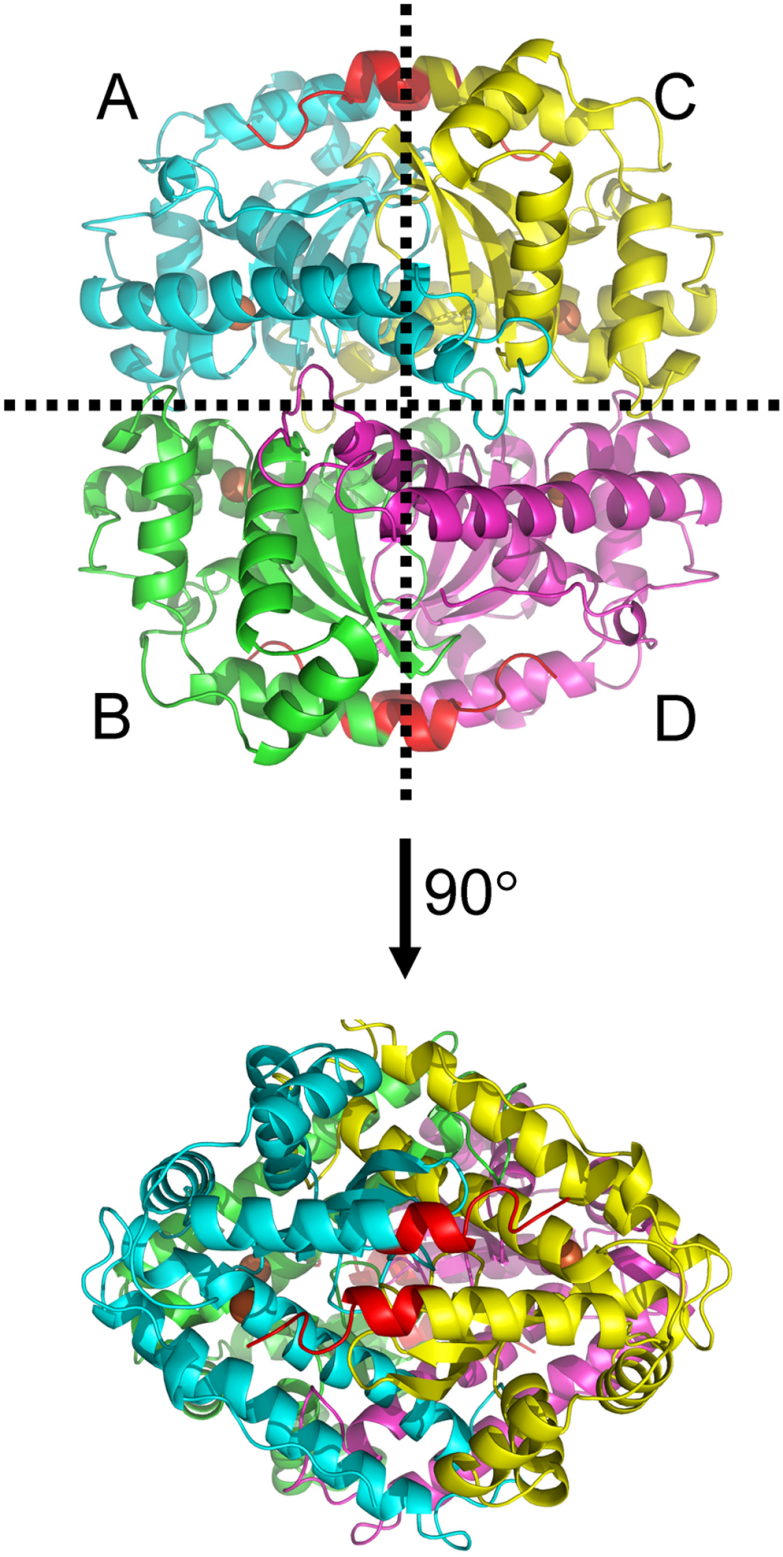
Crystal structure of ApSOD (PDB ID: 1COJ). The four subunits are distinguished in different colors. The coordinated Fe^2+^ ions are shown in the sphere model. The last 10 residues at the C-terminus are highlighted in red.

The structural basis for the formation of high-order oligomers remains unclear for tcSOD. It is worth noting that in the tetramer (Figure 8), the A/C subunit-interacting interface is far from the hydrophobic core and the catalytic centre, while the A/B interface contributes to the coordination of Fe^2+^ ions in the active sites of TcSOD or ApSOD [39], [40]. The fact that tcSOD high-order oligomers were fully active implied that the A/B interface was maintained in the large oligomers, while structural rearrangement was more likely to occur at the A/C interface. Consistent with this analysis, our mutational analysis indicated that the C-terminal helix, which is located at the A/C interface, might be involved in oligomerization. Meanwhile, cross-linking by DSP led to the dissociation of tcSOD tetramers into active dimers, suggesting that the high-order oligomers could be formed by using the active dimers as the building blocks. Domain-swapping is a well-defined mechanism to maintain the structure required for activity in the large oligomers or fibrils [50]. Considering that the last helices of subunits A and C interact with each other to stabilize the A/C interface in tetrameric Fe-SOD (Figure 8), it is possible that tcSOD also formed high-order oligomers via swapping of the C-terminal helix. This hypothesis is also consistent with the observations that the N- or C-terminal helix are usually involved in protein oligomerization through domain swapping or coiled-coil interactions in many hyperthermostable proteins [11], [51]–[54].

Although the detailed molecular mechanism remains elusive for tcSOD oligomerization, it is clear that conformational rearrangement is essential for the conversion from tetramers to high-order oligomers. The tetramer was quite stable at the room temperature, and high-order oligomers could be induced by heating at high temperatures (Figure 3). However, no significant changes in secondary and tertiary structures were observed by spectroscopic methods. Herein we showed that cross-linking is a very sensitive method to probe the subtle structural changes and/or conformational fluctuations. The inactivation induced by cross-linking using glutaraldehyde at high temperatures suggested that there was structural rearrangement occurred upon heat treatment. This structural rearrangement might be important to the optimal activity and/or oligomerization of tcSOD at high temperatures, which has also been observed in the other thermostable enzymes [14], [24], [25], [55]–[57]. More importantly, cross-linking experiments indicated that the amounts of the high-order oligomers of the WT TcSOD increased at high-temperatures, while no significant change was observed for M202. It is also worth noting that non-native aggregates could be characterized in the SEC profile for the cross-linked M202 at 80°C, but this is not the case for the WT. This implied that the C-terminal helix might be involved in the structural rearrangement induced by heat treatment, which is consistent with the proposal that the C-terminal helix was involved in the formation of high-order oligomers. When the ability of high-order oligomerization was removed by truncations, the mutant M202 was more prone to undergo the aggregation pathway at high temperatures.

**Figure 9:**
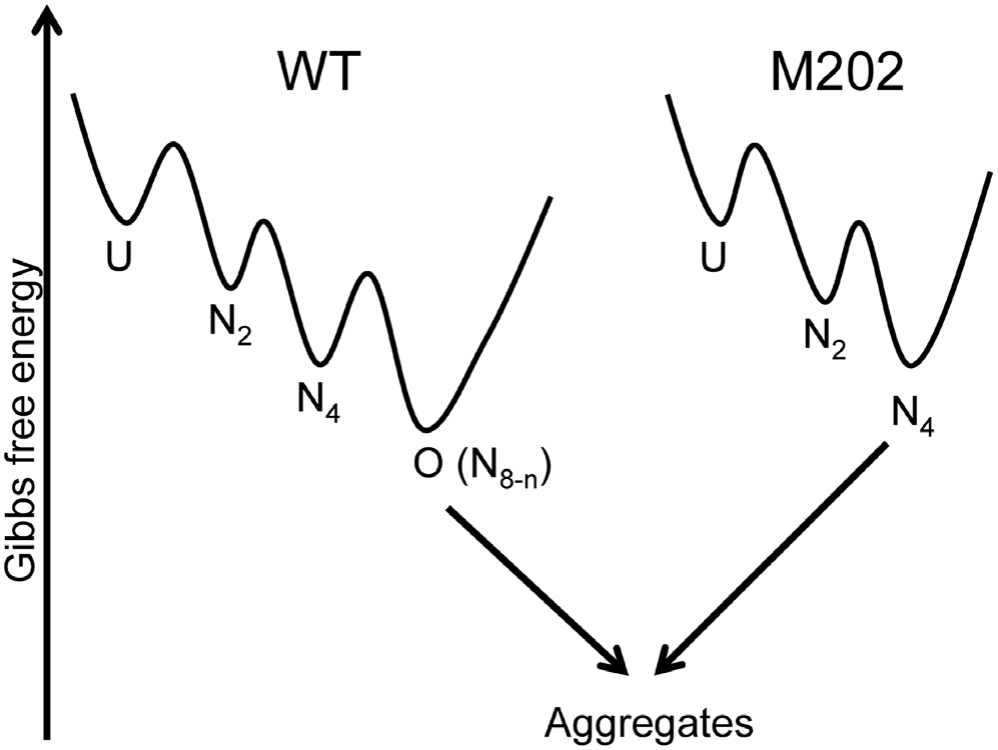
A proposed model for the role of active high-order oligomers in tcSOD hyperthermostability against off-pathway aggregation. The formation of active high-order oligomers is a reversible process, while the aggregation pathway is irreversible. The high-order oligomerization confers an additional energy gap between the native state and aggregates. The removals of the oligomerization ability by the M202 mutation facilitate the proteins to aggregate under extreme conditions.

In summary, the results herein suggested that the reversible conversion between the native tetramers and active high-order oligomers might provide a buffering system for tcSOD in fighting against the irreversible protein aggregation process (Figure 9). The formation of active high-order oligomers not only increases the energy gap between the native state and unfolded/aggregated state, but also provides the enzyme the potency to reproduce the predominant oligomeric forms since the core structures required for functions are well maintained in the high-order oligomers.

## Supporting Information

File S1 **Supporting information of this article with embedded Figures S1–S4.** Figure S1 shows the dependence of spectral characteristics and SEC elution volume on tcSOD concentration. Figure S2 shows the thermal stabilities of the WT and mutated tcSOD at 80°C. Figure S3 shows the thermal stability of tcSOD at 85°C evaluated by CD. Figure S4 shows the structural stability of tcSOD at 95°C evaluated by the maximum emission wavelength (*E*_max_) and CD signal.
(PDF)
