# Supplementary material for: Formation of High-Order Oligomers by a Hyperthemostable Fe-Superoxide Dismutase (tcSOD)
Source: PLoS One. 2014 Oct 14;9(10):e109657. doi: 10.1371/journal.pone.0109657 (PMC4196948; doi:10.1371/journal.pone.0109657)
Supplement: File S1 — Supporting information of this article with embedded Figures S1–S4. Figure S1 shows the dependence of spectral characteristics and SEC elution volume on tcSOD concentration. Figure S2 shows the thermal stabilities of the WT and mutated tcSOD at 80°C. Figure S3 shows the thermal stability of tcSOD at 85°C evaluated by CD. Figure S4 shows the structural stability of tcSOD at 95°C evaluated by the maximum emission wavelength (E max) and CD signal. (PDF) [file pone.0109657.s001.pdf]

Supporting Information File S1

Formation of high-order oligomers by a hyperthemostable Fe-superoxide dismutase (tcSOD)

Sha Wang[a,b], Zhi-Yang Dong[a]*, Yong-Bin Yan[b]*

[a] State Key Laboratory of Microbial Resources, Institute of Microbiology, Chinese Academy of Sciences, Beijing 100081, China

[b] State Key Laboratory of Biomembrane and Membrane Biotechnology, School of Life Sciences, Tsinghua University, Beijing 100084, China

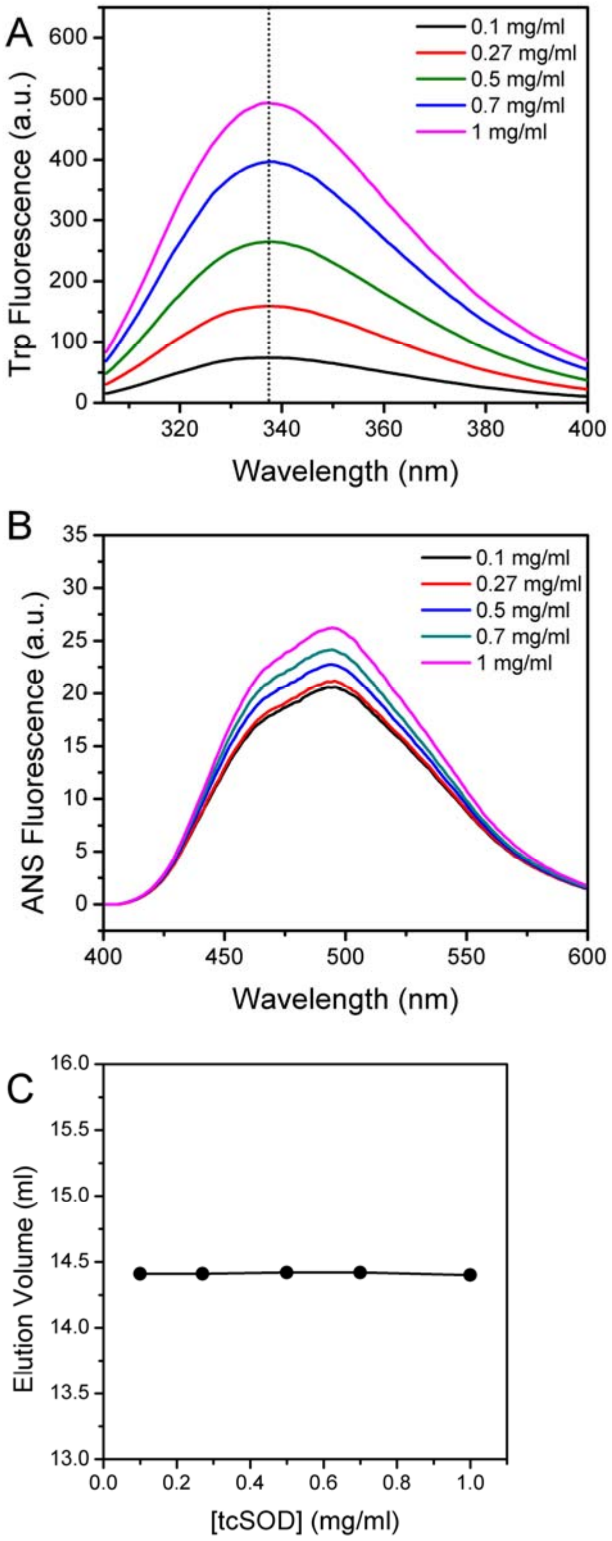

Figure S1. Dependence of spectral characteristics and SEC elution volume on tcSOD concentration. (A) Trp fluorescence excited at 295 nm. (B) ANS fluorescence excited at 380 nm. (C) Elution volume of the tetramer peak in the SEC profiles.

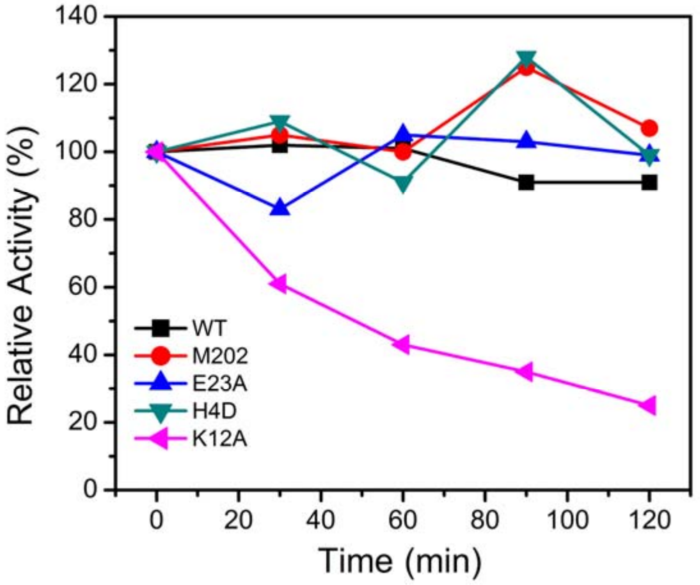

Figure S2. Thermal stabilities of the WT and mutated tcSOD at 80°C. The stabilities of the proteins at 95°C is presented in Figure 4C.

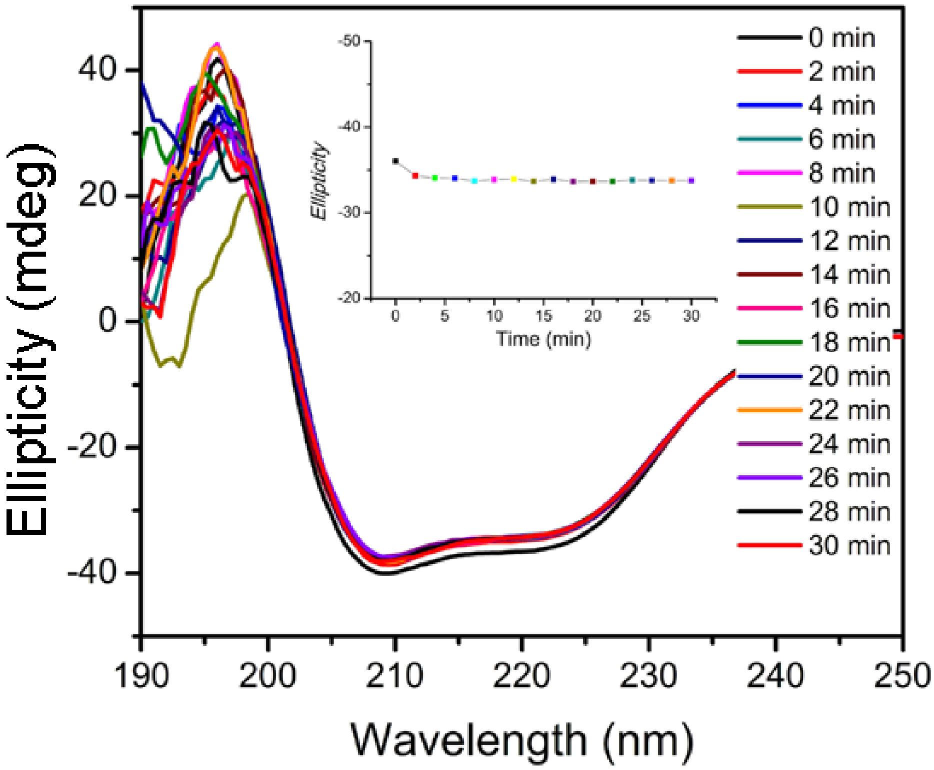

Figure S3. Thermal stability of tcSOD at 85°C evaluated by CD. The protein solution was heated at 85°C continuously and the CD spectra were collected every 2 min.

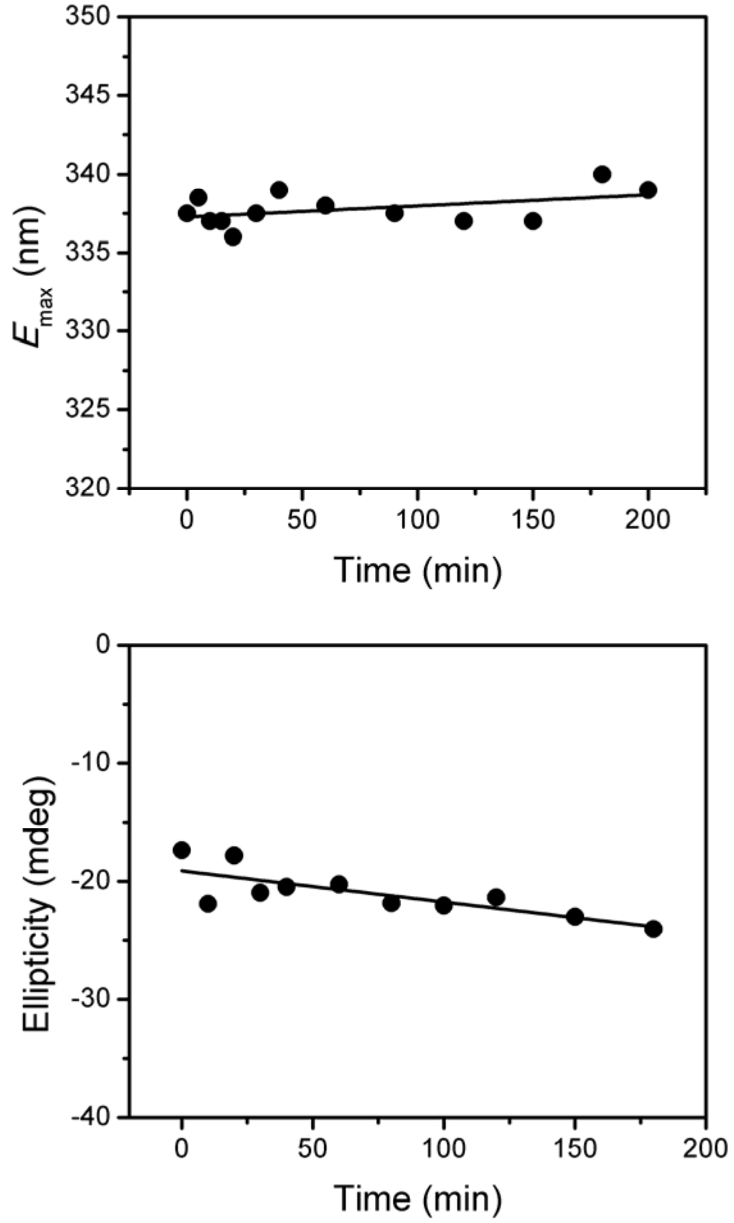

Figure S4. Structural stability of tcSOD at 95°C evaluated by the maximum emission wavelength (*E_{max}*) and CD signal. Due to the temperature range limitation of the water bath connected to the machine, the protein solutions was heated at 95°C for a given time and quenched on ice. Then the spectra were collected at ambient temperature.
